# Confidential, accessible point-of-care sexual health services to support the participation of key populations in biobehavioural surveys: Lessons for Papua New Guinea and other settings where reach of key populations is limited

**DOI:** 10.1371/journal.pone.0233026

**Published:** 2020-05-15

**Authors:** Angela Kelly-Hanku, Michelle Redman-MacLaren, Ruthy Boli-Neo, Somu Nosi, Sophie Ase, Herick Aeno, Joshua Nembari, Angelyn Amos, Josephine Gabuzzi, Martha Kupul, Barne Williie, Rebecca Narokobi, Parker Hou, Simon Pekon, John M. Kaldor, Steve G. Badman, Andrew J. Vallely, Avi J. Hakim

**Affiliations:** 1 Sexual and Reproductive Health Unit, Papua New Guinea Institute of Medical Research, Goroka, Eastern Highlands Province, Papua New Guinea; 2 Kirby Institute for Infection and Immunity in Society, UNSW Sydney, Sydney, New South Wales, Australia; 3 Division of Global HIV and TB, US Centers for Disease Control and Prevention, Atlanta, Georgia, United States of America; University of Pretoria, SOUTH AFRICA

## Abstract

To achieve the UNAIDS 90-90-90 targets at a national level, many countries must accelerate service coverage among key populations. To do this, key population programs have adopted methods similar to those used in respondent-driven sampling (RDS) to expand reach. A deeper understanding of factors from RDS surveys that enhance health service engagement can improve key population programs. To understand the in-depth lives of key populations, acceptance of expanded point-of-care biological testing and determine drivers of participation in RDS surveys, we conducted semi-structured interviews with 111 key population participants (12–65 years) were purposefully selected from six biobehavioral surveys (BBS) in three cities in Papua New Guinea. Key populations were female sex workers, men who have sex with men, and transgender women. Four reasons motivated individuals to participate in the BBS: peer referrals; private, confidential, and stigma-free study facilities; “one-stop shop” services that provided multiple tests and with same-day results, sexually transmitted infection treatment, and referrals; and the desire to know ones’ health status. Biobehavioral surveys, and programs offering key population services can incorporate the approach we used to facilitate key population engagement in the HIV cascade.

## Introduction

Despite several decades of intense global prevention and treatment efforts against HIV, curable sexually transmitted infections (STIs), and tuberculosis (TB), these diseases continue to cause an unacceptable burden of disease among key populations and their communities who also experience substantial social stigma and exclusion [1]. In 2017, 47% of all new HIV infections globally were among key populations [2] including sex workers; men who have sex with men; transgender persons; people who inject drugs; prisoners; and their partners. HIV incidence among key populations was 13 to 28 times higher than among other comparable groups [2]. Sexual behaviors that increase risk of HIV among key populations also increase risk for STIs and other bloodborne viruses [2]. If left untreated, these infections, including syphilis, gonorrhea, and chlamydia, can have adverse sexual and reproductive outcomes. As with other infectious diseases, TB disproportionately affects the most vulnerable: for example, people living with HIV are 21 times more likely to develop TB than HIV-negative individuals [3, 4]. TB accounts for one in three AIDS-related deaths, making TB the leading cause of death of HIV-positive individuals [1, 2].

Globally, there were 357 million new cases of curable STIs (chlamydia, gonorrhoea, syphilis and trichomoniasis) in 2012, of which 142 million were in the Western Pacific Region, including Papua New Guinea [5]. In addition to other STIs, Papua New Guinea has a concentrated HIV epidemic with a national adult HIV prevalence estimated at 0.9% [6]. The country’s response has become more focused on key populations [6], namely women and girls engaged in transactional sex (female sex workers [FSW]), men who have sex with men (MSM), and transgender women (TGW) [7]. For example, our recent key population biobehavioral survey (BBS) in Papua New Guinea, called *Kauntim mi tu* (translation, ‘Count me too’) (June 2016 –December 2017), showed that HIV infection rates among FSW are 11 to 19 times higher than among other adults [7, 8]. The estimated infection rates for MSM/TGW are 7 to 8 times higher than for other adults [7, 9].

With the global scale-up of ART, including in Papua New Guinea, some countries are on track to reach the UNAIDS 90-90-90 targets (in which 90% of HIV-positive people know their status, 90% of these people are on life-saving antiretroviral therapy [ART], and 90% of those receiving ART have suppressed HIV viral load), whereas others are not in a position to achieve these goals or to address the human rights issues hindering progress [2]. One of the reasons relates to poor coverage in HIV prevention among those most in need of prevention services, particularly key populations and children [2]. *Kauntim mi tu* has shown that Papua New Guinea is not on track to reach the 2020 UNAIDS 90-90-90 targets among FSW, MSM, and TGW [10].

BBS using respondent-driven sampling (RDS) [11] have achieved extensive reach [12–14]. Proportionally and relative to the time in the study sites, *Kauntim mi tu* reached a greater number of key population members than services specifically targeting the same groups. For example, *Poro Sapot*, the only dedicated HIV and sexual health service for key populations in operation at the time of our study, provided service including HIV testing to 453 people within key populations (370 were female sex workers and 83 were MSM or TGW) in Port Moresby in the last six months of 2015. Our study the following year, which also lasted six months, provided HIV testing services to 663 female sex workers and 390 men who have sex with men and transgender women. This success has generated interest among non-governmental and governmental organizations and donors in understanding why this BBS was so successful and applying these lessons in HIV programming into the future.

Drawing on qualitative insights provided as part of qualitative sub-study of the BBS designed to understand people’s lives in detail, we highlight factors for survey participation from the perspective of key populations. These insights can inform current and future programmatic responses for key populations locally in Papua New Guinea but can be applied to other settings where the reach of key populations has been limited, as well as future BBS.

## Materials and methods

*Kauntim mi tu* used RDS to recruit FSW and MSM and TGW into two separate BBS in three cities in Papua New Guinea: Port Moresby (June–October 2016) [15], Lae (January–June 2017) [16], and Mt. Hagen (August–December 2017) [17]. To take part in this study, FSW participants had to be born a biological female, to be aged ≥12 years, to have sold or exchanged vaginal sex in the past 6 months, to speak English or *Tok Pisin* (Melanesian pidgin), and to have a valid study coupon (i.e., invitation to join the survey given by a peer who has already participated in the survey). For MSM and TGW, participants had to be born a biological male, to be aged ≥12 years, have had oral or anal sex with another man within the past 6 months, to speak English or *Tok Pisin* (Melanesian pidgin), and to have a valid study coupon. For a more detailed discussion on the RDS method as it was used in this study see Hakim et al. [9]. For further details on issues with the eligibility age and waiver of parental / guardian consent see Ethics below.

The survey in *Kauntim mi tu* included demographics, sexual history and identity, condom use, family planning, stigma, social cohesion, violence, HIV knowledge, history of sexually transmitted diseases, and uptake of health services for example [18]. The two-item Patient Health Questionnaire (PHQ-2) was used to screen for depression [19]. In addition, study participants could opt in for several different point-of-care tests, including HIV, syphilis, hepatitis B virus, TB, and genital and anorectal gonorrhea and chlamydia. HIV-positive participants were provided with a CD4 T-cell test and point-of-care HIV viral load testing. Finally, a qualitative sub-study was undertaken with 60 FSW and 51 MSM and TGW BBS participants (age range 13–65 years). Due to low numbers of TGW in Papua New Guinea the community agreed to have sexually and gender diverse participants who were born male combined in the same study.

The qualitative study sought to understand—in context—the lives of the study participants. These participants were purposively selected and recruited, and the study team were provided with a matrix to identify FSW and MSM and TGW qualitative study participants at each of the three sites. The matrix was designed to ensure diversity across the populations to include people of different age groups, of different ethnic groups, and with different amounts of time living in the city. The matrix also called for the researchers to be mindful in recruiting people who reported diverse types of sexual behaviors and access to HIV prevention, testing, treatment, and care. The final criterion was that the participants should not have previously participated in another qualitative study by the Papua New Guinea Institute of Medical Research to ensure as much diversity as possible in the sample selected.

The interviews were conducted by trained Papua New Guinean researchers, almost always of the same sex at birth, who were also involved in the implementation of the survey in the larger study in which this qualitative work was embedded (RBN, SA, HA and JN). On average interviews took between 45 minutes to an hour and a half. Interviews were conducted in the same site as the overall study and in a space where participants could talk privately. A semi-structured interview guide designed in advance and approved by the relevant ethics committees.

The in-depth interviews were conducted in *Tok Pisin*, digitally audio recorded, transcribed verbatim, and then translated into English. All identifiable information was removed from the transcriptions, and pseudonyms were allocated to all participants and applied to participants in this and all manuscripts. All participants provided written informed consent and were reimbursed 20 Kina (US$6) for their participation.

### Data analysis

A coding framework was designed by a team of three Papua New Guinea–based researchers and was informed by the themes covered in the interview, including condom use, history of HIV testing, discrimination and violence, access to health services, sexual relationships, reasons for participating in the study, and experience of point-of-care testing. Using Nvivo (v11, QSR International) and the coding framework, these researchers initially coded the transcripts deductively. As thematic codes were identified, the original coding framework was inductively adapted to reflect themes that emerged from the data that had not been pre-identified. Using grounded theory methods [20] memoing was conducted throughout and informed the development of codes and categories to ensure data saturation and validity. Coding was reviewed and agreed upon by senior authors

### Ethics

There is a paucity of epidemiological and other data on young key populations, particularly those under the age of 18. WHO advocates for the need to urgently address the HIV epidemic among young key populations and adolescents as evident by their revised consolidated HIV guidelines for key populations, about which it has been said:

the guidelines highlight that it is urgent for countries to review their legal policies, initiate the provision of services as well as improve services, include adolescent and young key populations in developing acceptable services and offer opportunities for their meaningful inclusion in de- fining their HIV and health service needs, developing effective services and participating in research [21, pp.85-86].

*Kauntim mi tu* followed the global guidelines concerning parental / guardian waiver of consent for the inclusion of adolescents and young people in HIV biobehavioral surveys [18] which states:

In cases where survey participants are too young to give their own informed consent but obtaining informed consent from their parent is inappropriate or could cause harm to the participant, investigators can seek a waiver for parental informed consent… protecting a young person’s privacy may be better achieved by withholding notice of survey participation from the person’s parent or legal guardian. p.28

Moreover, according to Papua New Guinea’s HIV and AIDS Protection and Management ACT [22], in order to address the early age of sexual debut and mitigate against the adverse effects of age restrictions on HIV testing and services, people 12 years and older do not require parental consent to participate in HIV testing / sexual health services/programs. *Kauntim mi tu* was a study designed to improve and provide sexual health services.

*Kauntim mi tu* staff were trained by Save the Children in PNG on working with underage key populations including the need to ensure safety from harm and to identify and refer all sexually exploited persons, including all participants aged younger than 18 who reported selling or exchanging sex, to partner organizations experienced in providing social and other protective services.

The *Kauntim mi tu* study was approved by the Papua New Guinea National Department of Health’s Medical Research Advisory Committee, the Research Advisory Committee of the National AIDS Council Secretariat, the Papua New Guinea Institute of Medical Research’s Institutional Review Board, and the Human Research Ethics Committee at UNSW Sydney, Australia. The protocol was reviewed according to the Centers for Disease Control and Prevention’s (CDC) human research protection procedures and was determined to be research, but CDC involvement did not constitute engagement in human subjects’ research. CDC and UNSW Sydney investigators did not interact with participants or have access to identifiable data or specimens for research purposes. A letter of support was provided by *Friends Frangipani* and *Kapul Champion*, the peer-led civil societies for sex workers and for sexually diverse men and transgender people in Papua New Guinea.

## Results

Of the study participants (N = 2,955; 2,092 FSW and 863 MSM and TGW (of which 80 self-identified as a transgender woman)), 111 participated in in-depth interviews (FSW, 60; MSM and TGW, 51). The participants varied in terms of age, religious affiliation, place of origin, relationship status, HIV status, and sexual and gender identity (Table 1).

**Table 1:** Basic demographic details of participants interviewed as part of the qualitative sub-study of *Kauntim mi tu*.

	FSW (N = 60)	MSM & TGW (N = 51)
**Age, years**		
Range	13–45	18–65
Mean	27.7	30.2
**Place of recruitment**		
Port Moresby	18	22
Lae	20	19
Mt. Hagen	22	10
**Relationship status**		
Single / Never married	13	31
Married / Defacto to a man with one wife	10	1
Married to a man with multiple wives	3	-
Married with one wife	-	8
Married with multiple wives	-	3
Married with multiple transgender wives	-	1
Divorced/separated	30	7
Widow	4	-
**Religious affiliation**		
Seventh Day Adventist	14	11
Roman Catholic	9	6
Lutheran / United	5	8
Pentecostal / Other	12	8
Revival Church	5	1
Not reported	15	17
**Place of ethnic origin**		
Southern Region	6	7
Momase Region	5	10
Highlands Region	32	24
New Guinea Islands Region	2	1
Mixed heritage	12	8
Did not mention	3	1
**Sexual / Gender identity**		
Bisexual	-	11
Transgender Woman	-	4
Man who has sex with men (MSM)	-	8
Man of diverse sexual (MDS)		16
Gay man	-	4
Heterosexual man	-	8
**HIV positive**	22	5

Thematic analysis of the interview data revealed several important themes explaining why these participants participated in the *Kauntim mi tu* study. These insights offer important considerations for how to conduct BBS and key population health services more generally: peer referral; privacy, confidentiality, and a non-judgmental practice; diversity of tests and same-day results, and knowledge of health status. Of note, few of the narratives mentioned financial reimbursement as a motivating factor. Less tangible, but no less important, was the emotional response people shared about their involvement in the study, including feelings of excitement, happiness, and motivation.

### Peer referral

The recommendation of friends encouraged many of the participants to participate in the study. Pursuant to RDS methodology, study participants were asked to invite peers to join the study using a coupon, referred to by participants as ‘cards’. Cards were highly sought after because people were interested in testing; previous participants in some cases told their friends (‘bragged’) about the extensive nature of the tests offered. Eve, a 24-year-old FSW in Mt. Hagen, described her friend’s experience with the study:

[S]he came back with three cards and told me all about your establishment and work here… she encouraged us by saying that it would be good for us sex workers to go and check as the results were also being produced simultaneously. She bragged that she had done all her six tests already and handed me one of the cards, so I came here the very next day, that was yesterday, to check, and I have done it.

The opportunity to receive a referral card from a peer was highly valued by many participants. Lucas a 21-year-old MSM in Mt. Hagen explained that he was ‘lucky enough’ to receive a card from his friend. Others explained how they ‘excitedly accepted the offer’ (Monica, 25 years, FSW, Lae) and described the decision to be in the study: ‘an extraordinary kind of satisfaction filled me up, I just couldn’t sleep, my darling. I just couldn’t go to sleep’ (Tracey, 37 years, FSW, Lae).

Peer encouragement also supported participants’ willingness to be tested, many had never tested for HIV before. Nori, a 36-year-old MSM from Lae who had never attended a sexual health clinic before and who had never been tested for HIV, said, ‘I was scared, and I used never go. I never went, they might tell me that I am infected, and I don’t want to hear that. I was scared, and I did a lot of silly things, so I never went.’ A friend who had already participated in our study persuaded him to not focus on the things he had done that might have exposed him to HIV: ‘My friend said, “Forget it, come, there is a clinic. I will take you there, whether you are infected or what you will get treated, they will give you all the things you need. I am holding a card, so come we go”. So I said, “It’s ok, I follow my brother go and I will check.”‘

Julie, an 18-year-old FSW from Mt. Hagen who had symptoms of HIV, was similarly persuaded to participate in the study by a friend, her next-door neighbor who said:

“I want to give you a card so that you will go to that place,” she said. She said, “I could see that you were somebody else back then but now you look like somebody different, but anyway, I want you to go and simply get yourself tested because your health is important,” she said. But I said, “Let me die away, I am tired… I will remain like this, I don’t want to go,” I said. But she said, “Please, take this and go and access this service,” she said… When I arrived here I saw that there were a lot of women who came. So I felt at ease, and I said, “Hey, these women here and I have some similarities so I don’t think it’s a concern.” And when I came inside, I saw most of my friends in here as well….

Being encouraged by and/or going with a peer to the study site helped many participants. For example, Neeta a 24-year-old FSW who lives in Mt. Hagen (and had previously tested HIV-positive at a government clinic and had ceased HIV care and treatment) was approached by her neighbor who had already participated in *Kauntim mi tu*. Concerned for her health, Neeta described how her FSW peers were always inviting her to the clinic but that she would refuse saying, ‘I don’t want to, I don’t want to.’ On this occasion, her friend said, ‘I want to take you to a place,’ and rather than refusing, Neeta agreed for the first time: ‘I agreed, so I got changed and got ready on that day she made an appointment for me, and she also invited three other ladies as well, so we all came here together.’

For some participants, knowing people like themselves were attending made a positive impact on their decision to join the study. Kimi, a 40-year-old MSM in Port Moresby, explained he had never been sick, ‘so I never been interested to go and get myself tested until one of the boys said to me he had gone to this place and that gay men also went there. That was the time when I came here.’

After participating in the study with her friend, Neeta (above) described her excitement and relief at returning to HIV care and treatment:

I was so happy…I knew I had stopped taking ART, so I was waiting to die, just hanging around. But I was lucky that it was the Lord who made the way, so I had this chance of coming here to the study. That’s why I’ll now go back and take my medicine and go back to my normal form. I am excited.

The peer-driven nature of the referral process enhanced the sociability and thus accessibility of the study. Julie, an 18 year-old FSW from Mt. Hagen, described her experience of seeing friends at the study site: ‘I met them, so I felt happy, so we sat and chat, and I asked how they got invited, and they mentioned that they got it from women who passed it on to them, and I said, I did too. So, we were telling stories outside and then we came inside.’

Others let their friends join the study first and then attended once they were confident the experience would be a positive one, as explained by 30-year-old Negiso, a TGW from Lae:

Some of them said, “We’ll go and try first; then we’ll come back and let you know so you can go.” So, we let the older women go first, and they came here, they had a very good test, and when I went out, they told us that it was good…

### Privacy, confidentiality, and a non-judgmental practice

Because members of key populations are frequently discriminated against by family and the community, finding a safe space for health services where the staff are friendly, respectful, and non-judgmental was a dominant feature of people’s experience of *Kauntim mi tu*. The staff were described as respectful, with one participant explaining:

I was afraid when I first came here, but then as soon I went inside, I saw that they were all mothers there. They talked nicely to us and encouraged us not to feel ashamed of anything we thought, said, or had. (Fero, 22 years, MSM, Port Moresby).

Many participants felt positive about the study, with one stating that he had felt ‘ashamed’ and therefore never attended health clinics; however, with *Kauntim mi tu*, ‘I truly feel free and happy to be here’ (Maron, 26 years, MSM, Port Moresby).

In contrast to many previous experiences reported, the testing offered as part of the study was perceived as truly confidential, with Tyson describing it as a ‘silent test’ (31 years, MSM, Port Moresby). In addition, encouragement and practical support provided by staff at *Kauntim mi tu* were appreciated, as shared by Yaso, a 35-year-old MSM in Port Moresby: ‘At [CLINIC], you will only do testing and be given results, but here [they provide] counselling and encouragements, bus fare, and light refreshments.’ Others spoke of the unique and respectful service: ‘The service that you guys are providing is very nice, comfortable, and relaxing; everything is provided, tea, coffee. The atmosphere, the environment is friendly’ (Baku, 24 years, MSM, Port Moresby).

The quality of care provided by the staff at the clinics was also described by some participants. Gretel, a 26-year-old FSW from Mt. Hagen, reported that one of her friends, who had given her a card, said of her experience: ‘The health workers from the clinics and hospitals used to give us so many tablets… they do not know what kind of sickness we have, and they used to supply us with medicines only. But these people they will properly check your body.’ For others still, previous negative experiences accessing other health care services motivated them to participate in the study. Tyson, a 31-year-old MSM in Port Moresby, described his previous experiences:

I have gone to [CLINIC] for HIV testing three times. The first time I went, the doctor scolded me in public…I was ashamed, and I felt that it was not the right thing to do… So, when I heard from my brother that testing was also provided on site, I was grateful. I am happy to test because I’ve been having sex a lot of women and men and I don’t know what’s inside me or if there’s any infections that are developing in me. That’s why I agreed to come and at least test and see if I have any infections in me.

Tyson also described the service provided by the study: ‘There was no doctor to scold me or no nurse to tell me you are like this or that. I feel as if I’m home. I just had to go in there, checkup, and come out. Tea was available, and I am satisfied with the doctor.’ Comparing the service provided by the study to a local clinic, Neeta, a 24-year-old FSW in Mt. Hagen, shared that at her local clinic, they are kept ‘waiting until dark… They never give us the result quickly; that’s why we usually say, “Let them see it for themselves,” and we usually leave. With the study, it was easier to get the results, it was fast and easier.’

In addition to the quality of the staff and the non-judgmental services, participants also appreciated the confidentiality of testing and treatment. This confidentiality gave people more confidence to participate: ‘I sat down, and I saw that people moved from one station to another, and I thought this was going to be alright. It was confidential, so when my number was called, I went in. One thing I realized at first was that you guys were doing a great job’ (Avau, 29 years, MSM, Port Moresby).

Participants also cited the study sites, which were originally chosen with input from members of the community, as another reason for participating in the study. Study sites were secluded guest houses not associated with prior studies or any key population. Darius, a 24-year-old MSM in Port Moresby, described the seclusion and anonymity of the site: ‘The VCT sites they have their sign boards and what, and you know us Papua New Guineans, we always jump to conclusions.’

### “One-stop shop”: Diversity of tests and same-day results

As in many countries, Papua New Guinea’s health programs are organized vertically. For example, people go to sexual health clinics for HIV and STI services and TB clinics for TB services. People cannot access multiple services at one location where many services are provided. Papua New Guinea also has extremely limited laboratory capacity to test for and diagnose infections. Where testing is available people are required to return to the clinic to receive results and, if necessary, treatment. In most cases in Papua New Guinea, STIs are not screened for and treatment is based on syndromic management. This method misses many people who have an STI and are asymptomatic [23], and treating a disease with a known etiology is more effective than syndromic management, even for persons with symptoms.

Almost all participants expressed gratitude for the number of different tests offered as part of *Kauntim mi tu*. Excluding HIV testing (and CD4 t-cell test) and TB testing, participants had never had the opportunity to undergo these tests offered in this study (e.g., HIV viral load and urogenital and anorectal gonorrhea and chlamydia testing) or to undergo TB testing at the same time as STI or HIV testing. Mayo, a 43-year-old FSW in Lae, described her happiness in receiving a card from a peer and hearing that such a diverse range of tests were offered at one site:

They mentioned that your team here did a number of tests. That was what I heard when the information was being disseminated… when I came into this place, like when we go for HIV tests, we never get all the other types of tests. But when I came inside this place the other types of tests were provided by you. And that has made me so happy.

This was a sentiment shared by Francis, a 44-year-old MSM in Mt. Hagen, who reflected that same-day testing was ‘powerful.’ His experience of testing in his mining workplace, which provides good services compared with other health services in Papua New Guinea, were not comparable to his experience in the study: ‘To me it’s like wow. Do you understand? … It has never happened anywhere else.’

The scope and value of same-day testing was also described by Takai, a 25-year-old MSM in Lae. He appreciated that the study provided testing for other STIs: ‘I really like the services because you people provided testing for other sexual transmitted infections; if you have them you are at a very high risk of contracting HIV.’

Pamela, a 35-year-old FSW in Mt. Hagen, was similarly concerned about STIs. She was excited to be able to come and test; she recalled waking up early, taking a shower, skipping her breakfast, and coming straight to the study site to ensure she was one of the first there: ‘If the study leaves then oh no. They might leave anytime, but they have to get my [card] number first.’ She knew that she was HIV positive and was receiving ART, but she was worried that other STIs ‘might be inside,’ and while she may think of herself as a ‘normal woman’ without an STI, she might have one and not know. She came with the intent to know her STI status.

Tania, a 21-year-old FSW in Mt. Hagen, was motivated not only to be screened for STIs but also to measure her HIV viral load. Tania had been living with HIV for many years and was receiving treatment, but had never had her viral load tested because Papua New Guinea did not have a national HIV viral load program at time of the *Kauntim mi tu* and where it was being done in Port Moresby it was not at point-of-care. She felt that the treatment she was getting at her clinic was poor, saying that they did not even check her CD4 count. The chance then to determine her CD4 T-cell count and viral load was as Tania said, ‘my primary concern.’

Like others, Yaso, a 35-year-old MSM in Port Moresby, described the limitations of health services for key populations that focus only on HIV: ‘They only test for HIV, and if you are positive, they put you on treatment, and if you are not, then they supply you with condoms.’ What he liked about *Kauntim mi tu* was being tested for other infections such as STIs and TB. Being able to test for several infections was the same reason that Darius a 24-year-old MSM from Port Moresby was motivated to come. Usually ashamed to go to a clinic, Darius said, ‘It was those tests that prompted me to come.’

The opportunity to test for TB was well received. For example, Karu, a 22-year-old FSW in Lae who had already been diagnosed and treated with TB was worried still about TB and shared: ‘I was wondering about TB. I had been on TB medicine and I completed my dose and wondered whether I would still have TB. Like I was worried that I had TB. TB usually kills people. HIV I will live with for a long time, but TB usually kills people.’ Tracey, a 37-year-old FSW in Lae escribed testing for TB and other diseases and her reaction when a health professional told her the results: ‘When she told me I was negative to everything; I was so relieved. I was over the moon… If I were a dog with a tail, I’d be wagging my tail to show it. The kind of excitement I have today is so extraordinary.’

Participants appreciated not only the ability to test for STIs but also the ability to access treatment at the point of diagnosis: ‘The HIV result turned out negative. But as for the STIs, its result was positive, so yeah, I received treatment for that’ (Yomba, 20 years, MSM, Lae). The combination of same-day testing and treatment was powerful. The ability to obtain an accurate diagnosis and receive treatment drew Maron a 26-year-old MSM in Port Moresby to participate in the study. He explained that his friend shared a card and said ‘I would receive free treatment after my blood and samples were taken. Having said that, I was motivated to come.’ Yasa, a 33-year-old MSM in Lae, was diagnosed with TB as part of the study and was able to receive treatment immediately:

I came and found out properly, and I didn’t know that I had TB…Now you have me on medication, and I am on the supply, and I’m very happy.

Same-day results made some participants feel nervous, but the ability to obtain treatment immediately helped. Edna a 31-year-old FSW from Mt. Hagen explained, ‘I came here not feeling ashamed because I wanted to know my blood results that you people will give me. And I am just happy. But to take the blood and also to provide the results instantly, I was a bit scared. On the other hand, you people said that you were going to provide for the medication, I was happy.’ This range of feelings about testing and treatment at one site on the same day was a common experience for many study participants.

Much of the testing involved the use of self-collected urine samples and vaginal and anorectal swab samples, which was a new experience for many of the participants but was positively embraced. Most felt similarly to Gretel a 26-year-old FSW from Mt. Hagen, who was ‘just happy to give my specimen for testing.’ Saki, a 34-year-old MSM in Port Moresby, explained that although ‘it was a new thing,’ he was glad to ‘have gone through that.’ Eighteen-year-old Stanley from Mt. Hagen explained that as an MSM he was ‘very grateful’ for the anorectal swab, ‘because a lot of the times this is the only passage used by men to have sex with me, so I am very grateful.’ The need for extensive check-ups was described by Megusa, a 37-year-old TGW in Lae. She said, ‘They have to check us properly because we are having a lot of sex. We might have pus in our anus, even our mouth too, there might be brushes or frictions or whatever, as such.’ Ambo, a 21-year-old FSW in Lae, explained that she appreciated the urogenital and anorectal testing:

I liked it because it is true that we usually have anal sex as well as vaginal sex, therefore we usually use two parts of our body to have sex with, so when they explained that they will insert this plastic inside I simply accepted the idea, so I went and got the swab and collected the specimen from my anus and gave it to them. I did not mind.

Many participants felt comfortable using the swabs to collect samples, with some even feeling relief at participating in the extensive testing. The privacy afforded participants in the self-collection process was a positive aspect of the testing, as explained by Jacob, a 21-year-old MSM in Mt. Hagen:

I wasn’t scared or whatever. It was all because I had my privacy in there to test myself, that’s why I was not even concerned about anything. It’s to do with infections; that’s why I have to find out about the type of infection I might have. So I actually felt at ease with all these tests.

Not all participants felt so at ease. Some were uncertain, and others were ashamed, but there was little or no variation depending on study site or population. Julie, a 18-year-old FSW in Mt. Hagen, explained, ‘I was in two minds about it, but then I started to think that since I have never visited a health facility, they are doing it purposely for my health; that’s why it’s all right.’ Rocky, a 25-year-old MSM from Port Moresby, stated, ‘It was okay; it was odd but because the fact that I am doing it myself it’s okay, but then I was thinking like what if there’s any shit on it or yea. That’s the thing.’ Maria, a 25-year-old FSW in Port Moresby, explained how she navigated the challenge of this new method with the support of the clinician:

“Sister, how do I do it?” and she said, “You put it, you will go into the toilet and you will put one into the vagina and the other one into the anus.” I’d never experienced this, so it was my first time… I went in, and I stood, I looked at the instructions on the wall, and then I came back outside… I thought that it would be a bit difficult or what, but it was alright.

Any fear or nervousness of self-collection was countered by knowing about potential infections, as Mofa, a 20-year-old FSW from Lae, explained:

I thought like, well, the anal, like while I pushed the stick through, I thought it might go right inside and I was already afraid. Even though I was afraid, I thought my life was important, so in case I had some disease, I continued to push through. I turned around, and I did it. I returned the test to her.

### Knowing health status

While people wanted to know their health status regarding the infections included in *Kauntim mi tu*, the motivations varied, often rather significantly. Tamox an FSW in Port Moresby (age not known) said she was excited to participate in the study and know if her body had any infections. Mike, a 26-year-old MSM in Port Moresby, said that he had previously never ‘respected’ his body and that this study provided an opportunity to ‘come to find out and know about my inside, and the outside, of my body.’ Also referring to her body, Ato, a 30-year-old FSW in Lae, shared that she needed to tell the researchers her story and that they must ‘check what kind of sickness I have in my body, they must see it themselves and tell me. I must know that I have this disease.’

Nineteen-year-old Lavinia, from Mt. Hagen, said that as a FSW it was ‘helpful’ to know and understand “about some of the diseases in our body” because previously participants were not able to test for potential asymptomatic infections, such as microbial STIs. Because a person can live with HIV without knowing they are positive, 17-year-old Betty, an FSW in Lae, decided to test for HIV because she wanted to know if it was ‘hiding away in my body.’ Ambo, a 21-year-old FSW in Lae, described a time when she refused unprotected sex with a boy from the street saying, ‘You don’t know about my body, and I don’t know about yours.’ Ambo was ‘scared to go and stand to listen to awareness’ about HIV and STIs, but her pregnancy motivated her to check her status during the study: ‘I want to check myself whether I am healthy, or whether I am exposed.’ The testing provided as part of the study, which was not available elsewhere, enabled participants to know their health status in a space they identified as safe.

While some study participants already knew that they were HIV positive or had previously tested negative, still others had never been tested for HIV. Raka, a 31-year-old MSM from Port Moresby, shared,

In my entire life, I haven’t gone and done one HIV testing or counselling. I have not gone, this is my first time… I heard about this, and I was happy, so I grabbed this card from them, and I said, “I’m going.” Now I’m middle-aged, and I haven’t tested, so I must go. I must know about my status, whether my life is all right, or which part of my life is good or bad. I must check out and balance my body and stay.

Many sex workers were aware of their risk of STIs, including HIV, and wanted to know whether they were infected. ‘Women who usually go around selling sex, they used to get this kind of sickness. All kinds of STIs like HIV, gonorrhea, such sickness,’ was how 42-year old Sandra, an FSW from Port Moresby, explained the risk. In Mt. Hagen 26-year-old Gretel felt ‘scared’ that as a FSW she may be in infected with something, while 17-year-old Betty, also an FSW but in Lae said, ‘You might continue to do that [sex work] and end up contracting AIDS, so remember: AIDS does not have any cure; therefore, it can take your life anywhere on the streets.’ Also an FSW from Mt. Hagen, 33-year-old Judy was similarly motivated to be teste because she regularly sold sex.

Some participants were motivated to be in the study and get tested not only because of their behavior, but also because of the behavior of their partner’s partners. Bobby, a 36-year-old MSM in Lae shared, ‘The reason behind me doing these tests was that I was involved [had sex] with my [sex] partners, both men and women, but also those partners have other partners.’ Lucas, a 21-year-old MSM in Mt. Hagen, has a peer who is HIV-positive, and this motivated him to learn about his own HIV status:

The reason why I came here for testing is that one of my peers was infected with HIV virus, so that was eye opening, and it’s better to know of our status… when we were aware of our peer’s status, we recalled the times we had sex together, and the woman whom he usually goes out with is also our sexual partner, so we were so afraid that we might be infected as well.

Thirty-one-year-old Raka, an MSM in Port Moresby, described the power of testing and finding out he was healthy and uninfected: ‘I feel confident that I am a full human being living now. I don’t feel that I am a half human being, or I don’t have bits and pieces.’ Despite varying motivations, people participated in the study because they wanted to know their health status regarding the infections included in *Kauntim mi tu*.

## Discussion

This qualitative exploration of motivations for key population participants in a large biobehavioral survey in Papua New Guinea provides critical insights for both researchers and services providers. In this study, we reached more people than service providers have reached and in a very short period at each of the study sites. Our findings provide a nuanced understanding of our success in recruitment in such a short period. We identified four themes that encouraged participation: peer referral; privacy, confidentiality, and a non-judgmental practice; diversity of tests and same-day results; and knowledge of health status. The expanded use of point-of-care testing not only was acceptable but also motivated many to participate in the study.

Trusting peers and their description of their experience at the study site gave people confidence in the staff and the services offered. This trust was based on a truly confidential experience where they knew their names, results, and stories would not be shared with others. *Kauntim mi tu* provided a physical space where people could feel safe to be themselves and tell their stories, sometimes for the first time. This space was made more comfortable with the availability of refreshments, lounges (rather than rigid upright chairs), films, and magazines, spaces to relax and be social. Space also was provided for participants to smoke and chew *buai* (betel nut). We worked hard to ensure that the needs of the participants were met and that their environment was enjoyable and conducive for them to stay and to refer their peers. Recently, attention is being drawn to the importance of the health clinic, particularly sexual health clinics, to be engaging, enjoyable, and even pleasurable [24]. The study spaces were more of a ‘leisure space’ than a medical one [24].

In addition to creating a space that was enjoyable and safe, we provided high-quality health services unavailable elsewhere. Our study staff neither shamed nor stigmatized participants because of their sexual behaviors, including anal sex and the need for anorectal STI testing. Our study staff celebrated our participants as human beings and normalized their practices. Before study initiation, study staff completed rigorous training on initiating conversations about STIs, including anorectal STIs, and making participants feel comfortable to discuss these issues as well as any fears or concerns they might have in providing specimens with fecal matter on the swab. The staff’s efforts to destigmatize and de-escalate any fear likely contributed to an unexpectedly high uptake of anorectal STI testing among all populations. Incentives in *Kauntim mi tu* were not calculated on the number of tests participants consented to. Therefore, we do not believe that uptake on anorectal STI testing was driven by financial interests.

De-escalation and destigmatization in sexual health services are key to making sexual health clinic spaces accessible, pleasurable, and enjoyable [24], enabling expanded reach and coverage, as demonstrated in *Kauntim mi tu*. Without addressing the barriers to access and reach for key populations in their relevant settings, and providing quality services, as exemplified by *Kauntim mi tu*, members of key populations will not access health services. When such reach and coverage is achieved it is then possible to improve both the lives of marginalized people and their communities but also support a country in its efforts to achieve the UNAIDS 90-90-90 goals.

To ensure that the study was welcoming and acceptable to the community, we worked in partnership with the FSW, MSM, and TGW communities to name the study, to identify the unique objects used for size estimation activities at each study site implemented prior to the study [25], to select study sites, and to identify trusted members of the community to take on key roles in the study team. This, in conjunction with the quality services and the respect of staff enabled us to show that we appreciate and understand how fear, negativity, stigma, and lack of privacy dissuade members of key populations from attending health clinics, especially those who are not reached by standard services.

Our approach, which allowed us to recruit and reach so many members of key populations, often deemed hard to reach, across the three cities, can guide other health services and outreach programs. We designed *Kauntim mi tu* in close consultation with members of key populations and implemented the study in a genuine partnership with them and their civil society organizations. Members of these populations were also employed in strategic positions within the study team. We listened and respected the insights and contributions of key population members. We also provided a space where participants were respected and were encouraged not to feel fear or shame about HIV or STIs, particularly anal STIs, which are largely ignored in sexual health clinics in Papua New Guinea. Although *Kauntim mi tu* was a cross-sectional survey, the lessons from its successful implementation and wide reach are important to consider and can be adapted and applied to ongoing routine and other sexual health services for these marginalized populations.

For a qualitative study about key populations that is concerned with in-depth, nuanced understandings from an emic perspective, a sample of 111 participants is more than sufficient. Qualitative research seeks data saturation whereby no new information is being generated by additional interviews. We reached this saturation suggesting that our findings on factors that affected participation are likely to reflect the wider sample of key populations who participated in the *Kauntim mi tu*. The sample was purposely sampled to ensure diversity of participants and therefore reduce bias by only sampling for example, people of a particular age or HIV testing history. While we do not concern ourselves with generalizability in the way the quantitative research does, we are confident that the narratives collected are sufficiently different and similar to allow for interpretive approaches to the phenomena at hand.

## Conclusion

By listening carefully to the community’s needs, we designed a study that not only provides critical behavioral and biological information to the national government but also provided key populations with exemplary, confidential health services that improves their lives. We helped empower study participants to have greater control and autonomy over their lives, which is a core principal in Papua New Guinea’s STI and HIV strategy for the next five years. Our findings from these qualitative interviews with study participants can help improve sustainable key population services beyond a research setting, and beyond Papua New Guinea to other low- to middle-income countries with concentrated epidemics.
